# Long-term safety and efficacy of a foldable iris-fixated phakic intraocular lens for the correction of myopia

**DOI:** 10.1007/s10792-023-02850-8

**Published:** 2023-08-17

**Authors:** Mariano Royo, Ángel Jiménez, David P. Piñero

**Affiliations:** 1 Department of Ophthalmology, San Rafael Hospital, Madrid, Spain; 2 Instituto Oftalmológico de Madrid, Madrid, Spain; 3 Department of Optics, Pharmacology and Anatomy, University of Alicante, Crta San Vicente del Raspeig S/N 03016, San Vicente del Raspeig Alicante, Alicante, Spain

**Keywords:** Phakic intraocular lens, Artiflex, Myopia, Anterior chamber depth, Intraocular pressure, Corneal endothelial cell density

## Abstract

**Purpose:**

To analyze and report the long-term outcomes in terms of efficacy and safety of eyes implanted with the spherical version of a foldable iris-fixated phakic intraocular lens (pIOL) for the correction of myopia.

**Methods:**

Retrospective analysis of the results of 56 eyes of 32 patients (age, 19–45 years) who underwent implantation of the spherical model of the Artiflex pIOL (Ophtec B.V., Groningen, The Netherlands) for the correction of myopia. Visual, refractive, biometric, intraocular pressure (IOP) and corneal endothelial changes were evaluated during a long-term follow-up: 2, 7, 10 and 12 years for more than 50, 30, 20 and 10 eyes, respectively.

**Results:**

At 4 weeks postoperatively, a significant reduction of manifest sphere and spherical equivalent (SE), with a significant improvement of uncorrected distance visual acuity were found (all *p* < 0.001). No significant changes were found during the rest of follow-up in sphere (*p *≥ 0.072). The percentage of eyes with SE within ± 1.00 D was over 83% during the whole follow-up. A non-significant trend to IOP increase was observed at 4 weeks postoperatively (*p* = 0.530), with a significant reduction at 1 year after (*p* = 0.039) and no significant changes during the rest of follow-up (*p* = 0.180). There was a significant reduction of anterior chamber depth at 4 weeks after surgery (*p* < 0.001), with no significant changes during the following 9 years of follow-up (*p* = 0.118). However, an additional significant decrease of this parameter was observed between 10 and 13 years after surgery (*p* = 0.027). Mean endothelial cell loss changed from 2.01 ± 4.49% at 4 weeks after surgery to 9.11 ± 2.24% at the end of the follow-up. No complications were reported during the follow-up.

**Conclusions:**

Myopia correction with the Artiflex pIOL is an effective and safe procedure in the long term.

## Introduction

Iris-fixated phakic intraocular lenses (pIOLs) have been available for over 25 years, with extensive research reporting the safety and efficacy of these implants for the correction of myopia [1–6]. Initially, this type of pIOL was made of rigid material which supposed the need for a large corneal incision for its implantation, with the potential of induction of some unpredictable astigmatic changes with such incision [7]. For this reason, foldable iris-fixated pIOLs were developed which could be inserted into the eye through a small incision, allowing a better control of corneal astigmatism. These foldable iris-fixated pIOLs have shown to provide an efficacious correction of myopia [1–4, 6, 8–15]. Regarding safety, as happened with the rigid version of this type of pIOL, several studies have reported good levels of preservation of corneal endothelium and minimal incidence of complications in the short, medium and long-term [1–4, 6, 8–15]. The longest series reported to this date evaluating the outcomes of foldable iris-fixated pIOLs were reported by our research group (8 years of follow-up) [8], by Papa-Vettorazzi et al. [2] (≥ 10 years of follow-up), van Rijn et al. [3] (15 years of follow-up, hyperopia model) and by Marta et al. (15 year of follow-up) [1]. Our research group [8] found a total endothelial corneal density (ECD) loss of 4.8% at 8 years after implantation of the spherical version of the commercially available foldable iris-fixated pIOL Artiflex (Ophtec B.V., Groningen, The Netherlands), whereas Papa-Vettorazzi et al. [2] reported a mean ECD loss of 12.2 ± 12.5% for a longer follow-up.

The aim of the current study was to analyze and report the long-term follow-up outcomes in terms of efficacy and safety of eyes implanted with the spherical version of the foldable iris-fixated pIOL Artiflex for the correction of myopia.

## Methods

### Patients

This study was a retrospective analysis of the results of patients who underwent implantation of the spherical model of the Artiflex pIOL for the correction of myopia from 2008 to 2022 at Madrid Ophthalmological Institute. Specifically, data from a total of 56 eyes of 32 patients were collected and analysed. Patients included had a variable follow-up: 2, 7, 10 and 12 years for more than 50, 30, 20 and 10 eyes, respectively. Signed informed consent was obtained from all patients before the operation, indicating that the clinical data obtained was going to be used anonymously for future analyses. The study adhered to the tenets of the Declaration of Helsinki, and it was approved by the clinic medical ethics committee.

Inclusion criteria for the study were the eligibility criteria for this type of surgery: central anterior chamber depth of 2.7 mm or more (measured from the endothelium), minimum ECD of 2200 cells/mm [2], stable refraction (at least 1 year), and mesopic pupil size of 6.0 mm or less. Contact lens use was recommended to be stopped at least 1 week before the preoperative visit. If signs compatible with corneal warpage were detected in this first visit, examinations were repeated weekly until confirming that stable results were obtained in at least two consecutive visits. Exclusion criteria included any active ocular pathology, including retinal disorders and corneal dystrophies, previous ocular surgery, aniridia or iris atrophy, pregnancy, diabetes, IOP over 21 mm Hg, age below 18 years and any systemic disease that could increase operative risk or confound the outcomes.

### Clinical protocol

A very strict clinical protocol was followed since 2008 in all patients implanted with the pIOL evaluated, with a complete preoperative examination including anamnesis, manifest and cycloplegic refraction, measurement of uncorrected (UDVA) and corrected distance visual acuity (CDVA), slit lamp biomicroscopy, corneal topography (Pentacam, Oculus Optikgeräte GmbH, Wetzlar, Germany), optical biometry (IOL-Master, Carl Zeiss Meditec AG, Jena, Germany), endothelial cell count using a noncontact specular microscope (EM-3000, Tomey, Nagoya, Japan), Goldman tonometry, and fundus evaluation.

Patients were scheduled for evaluation the day after surgery, at 4–5 weeks and 6 months postoperatively and then each year to evaluate the long-term stability of the outcomes obtained. The day after surgery, the integrity of the anterior chamber was evaluated by means of slit lamp biomicroscopy, with additional measurement of UDVA and IOP. In the rest of visits, the same examination protocol was followed that included measurement of UDVA and CDVA, manifest refraction, biomicroscopy, tonometry, corneal topography and endothelial cell count.

### Surgical protocol

All surgeries were performed by the same experienced surgeon (MR) using either peribulbar or retrobulbar anaesthesia depending on surgeon’s preference. Miotic drops were instilled in all patients before operation. First, a corneal incision of 3.2 mm at the 12 o’clock position was performed for the implantation of the pIOL. Two stab incisions were located at 10 and 2 o’clock positions. Then, viscoelastic was injected into the anterior chamber and the pIOL then inserted. Afterwards, the lens was fixated in the iris by the haptics claws. Once the pIOL was positioned, an iridotomy was made to facilitate aqueous humour flow, and viscoelastic material was removed and replaced for balanced saline solution. The corneal incision was then hydrated for its sealing.

### Phakic intraocular lens model

The study was carried out using the Artiflex Myopia pIOL (Ophtec B.V., Groningen, The Netherlands). This lens has a 6-mm diameter flexible optical part made of silicone and PMMA rigid haptics. It has a total diameter of 8.5 mm. This pIOL is available in optical powers from −2.0 to −14.5 D. The selection of the pIOL power to implant was performed according to the manufacturer, entering the refractive power, ACD and keratometry data in the Van der Heijde formula [16].

### Data analysis

Statistical data analysis was performed using the software SPSS version 22.0 for Windows (SPSS, Chicago, Illinois, USA). Normality of all data distributions was evaluated by means of the Kolmogorov–Smirnov test. For the analysis of the statistical significance of differences between consecutive visits the repeated-measures analysis of variance (ANOVA) was applied for parametric data, whereas the Friedman’s test was used for non-parametric data. Post-hoc comparisons were performed with the Bonferroni test for parametric samples and with the Wilcoxon test (with Bonferroni adjustment) for non-parametric data. A *p*-value of less than 0.05 was considered as statistically significant.

Concerning refraction notation, the power vector method described by Thibos and Horner was used for converting the preoperative and postoperative spherocylindrical refractions into vector notation [17]. With this method, any spherocylindrical refractive error can be expressed by a vector in a 3-dimensional dioptric space (M or SE, J_0_, J_45_), being the length of this vector a measure of the overall blurring strength (B) of a spherocylindrical refractive error. M is the spherical equivalent refraction, and J_0_ and J_45_ are the 2 Jackson crossed cylinders equivalent to the conventional cylinder. Specifically, manifest refractions in conventional script notation (S [sphere], C [cylinder], φ [axis]) were converted to power vector coordinates and overall blurring strength using the following formulas: M or SE = S + C/2; J_0_ = (−C/2) cos(2φ); J_45_ = (−C/2) sin(2φ); B = (M^2^ + J_0_^2^ + J_45_^2^)^1/2^.

Finally, the standard graphs for reporting the outcomes of refractive surgery were adopted to present the results of the current series, according to the procedure described by Reinstein et al. [18].

## Results

Data from a total of 56 eyes of 32 patients with mean age of 27.6 years were analysed. The follow-up was not completed by all patients, with a total of 54, 51, 47, 39, 37, 36, 34, 27, 27, 24, 17, 11, and 6 eyes followed for 1, 2, 3, 4, 5, 6, 7, 8, 9, 10, 11, 12 and 13 years, respectively. Table 1 summarizes all demographic and preoperative data.

**Table 1:** Demographic and preoperative characteristics of the evaluated sample

Parameter	Mean (SD)	Median (range)
Age (years)	27.6 (6.9)	27.0 (19 to 45)
Gender	17 males (53.1%), 15 females (46.9%)	
Eye	26 right eyes (46.4%), 30 left eyes (53.6%)	
LogMAR UDVA	1.94 (0.23)	2.00 (1.00 to 2.00)
Sphere (D)	− 8.65 (3.36)	− 7.88 (− 18.00 to − 2.50)
Cylinder (D)	− 0.66 (0.76)	− 0.50 (− 3.00 to 0.00)
SE (D)	− 8.97 (3.49)	− 8.19 (− 18.25 to − 2.87)
J_0_ (D)	0.15 (0.39)	0.00 (− 0.74 to 1.30)
J_45_ (D)	− 0.03 (0.28)	0.00 (− 0.80 to 0.75)
B (D)	8.99 (3.49)	8.21 (2.90 to 18.25)
LogMAR CDVA	0.06 (0.09)	0.00 (0.00 to 0.30)
AXL (mm)	26.87 (1.65)	26.71 (23.66 to 31.82)
ACD (mm)	3.35 (0.24)	3.31 (2.87 to 3.85)
K1 (D)	43.79 (1.77)	43.90 (38.50 to 48.30)
K2 (D)	44.47 (1.54)	44.75 (41.00 to 47.40)
Power of the lens implanted (D)	− 9.09 (2.85)	− 8.50 (− 14.50 to − 3.50)

SD: standard deviation; D: diopters; UDVA: uncorrected distance visual acuity; SE: spherical equivalent; J_0_ and J_45_: power vectors of refractive astigmatism; B: overall blur strength; CDVA: corrected distance visual acuity; AXL: axial length; ACD: anterior chamber depth; K1: flattest keratometric reading; K2: steepest keratometric reading

### Visual and refractive changes

**Fig. 1:**
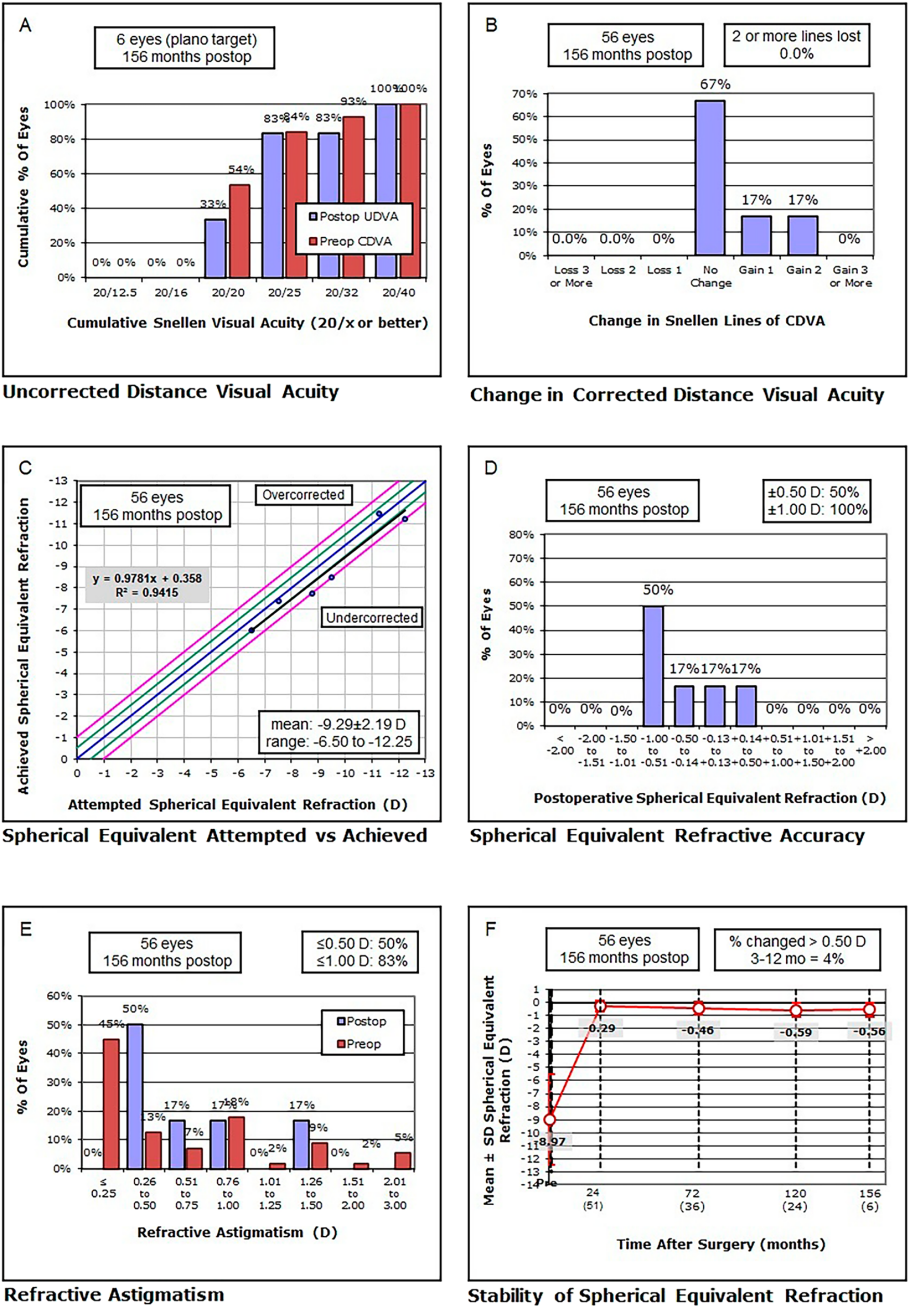
Standard graphs for reporting the visual and refractive outcomes following the guidelines described by Reinstein et al.[18]

Table 2 and Fig. 1 summarizes the visual and refractive data of the sample evaluated. Significant changes during the follow-up were found in all parameters (*p* ≤ 0.045), except J_45_ (*p* = 0.058) and CDVA (*p* = 0.149). At 4 weeks after surgery, significant reductions of manifest sphere, J_0_, SE and B (all *p* < 0.001) combined with a significant improvement of UDVA (*p* < 0.001) were found. No significant changes were found during the rest of follow-up in sphere (*p* = 0.395), SE (*p* = 0.072), and UDVA (*p* = 0.317). However, significant changes were found from the 4-week postoperative visit to the 13-year visit in J_0_ (*p* = 0.028) and B (*p* = 0.028). Regarding manifest cylinder, no significant changes were found at 4 weeks after surgery (*p* = 0.435), but the change in this parameter from 1 to 11 years after surgery did reach statistical significance (*p* = 0.045). No significant changes in manifest cylinder were detected afterwards (11–13 years after surgery) (*p* = 0.317).

**Table 2:** Summary of postoperative visual and refractive data in the sample evaluated

Mean (SD) Median (Range)	N	LogMAR UDVA	Sphere (D)	Cylinder (D)	SE (D)	J_0_ (D)	J_45_ (D)	B (D)	CDVA
Preoperative	56	1.94 (0.23) 2.00 (1.00 to 2.00)	− 8.65 (3.36) − 7.88 (− 18.00 to − 2.50)	− 0.66 (0.76) − 0.50 (− 3.00 to 0.00)	− 8.97 (3.49) − 8.19 (− 18.25 to − 2.87)	0.15 (0.39) 0.00 (− 0.74 to 1.30)	− 0.03 (0.28) 0.00 (− 0.80 to 0.75)	8.99 (3.49) 8.21 (2.90 to 18.25)	0.06 (0.09) 0.00 (0.00 to 0.30)
4 weeks	56	0.05 (0.10) 0.00 (0.00 to 0.52)	− 0.05 (0.34) 0.00 (− 1.25 to 0.75)	− 0.46 (0.49) − 0.50 (− 3.00 to 0.00)	− 0.28 (0.38) − 0.25 (− 1.50 to 0.25)	− 0.08 (0.20) 0.00 (− 0.49 to 0.32)	− 0.06 (0.25) 0.00 (− 0.65 to 1.48)	0.41 (0.41) 0.35 (0.00 to 2.12)	0.03 (0.08) 0.00 (0.00 to 0.30)
6 months	54	0.05 (0.10) 0.00 (0.00 to 0.52)	− 0.02 (0.26) 0.00 (− 0.75 to 0.75)	− 0.51 (0.63) − 0.50 (− 3.00 to 0.00)	− 0.27 (0.42) − 0.25 (− 1.50 to 0.25)	− 0.05 (0.23) 0.00 (− 0.74 to 0.96)	− 0.06 (0.30) 0.00 (− 1.15 to 1.48)	0.40 (0.49) 0.25 (0.00 to 2.32)	0.03 (0.07) 0.00 (0.00 to 0.30)
1 year	54	0.04 (0.08) 0.00 (0.00 to 0.40)	− 0.01 (0.25) 0.00 (− 0.75 to 0.75)	− 0.47 (0.49) − 0.50 (− 3.00 to 0.00)	− 0.25 (0.36) − 0.25 (− 1.50 to 0.25)	− 0.08 (0.25) − 0.02 (− 0.96 to 0.70)	− 0.09 (0.19) 0.00 (− 1.15 to 0.12)	0.41 (0.38) 0.35 (0.00 to 2.12)	0.03 (0.06) 0.00 (0.00 to 0.30)
2 years	51	0.05 (0.09) 0.00 (0.00 to 0.40)	− 0.03 (0.29) 0.00 (− 1.00 to 1.00)	− 0.51 (0.51) − 0.50 (− 3.00 to 0.00)	− 0.29 (0.40) − 0.25 (− 1.50 to 0.25)	− 0.04 (0.27) 0.00 (− 0.96 to 0.70)	− 0.10 (0.22) 0.00 (− 1.15 to 0.32)	0.45 (0.41) 0.35 (0.00 to 2.12)	0.02 (0.06) 0.00 (0.00 to 0.30)
3 years	47	0.06 (0.09) 0.00 (0.00 to 0.40)	− 0.06 (0.34) 0.00 (− 1.00 to 1.00)	− 0.52 (0.58) − 0.50 (− 3.00 to 0.00)	− 0.33 (0.44) − 0.25 (− 1.50 to 0.25)	− 0.05 (0.29) 0.00 (− 0.96 to 0.77)	− 0.09 (0.24) 0.00 (− 1.15 to 0.64)	0.49 (0.46) 0.35 (0.00 to 2.12)	0.03 (0.06) 0.00 (0.00 to 0.30)
4 years	39	0.07 (0.10) 0.00 (0.00 to 0.40)	− 0.09 (0.36) 0.00 (− 1.00 to 1.00)	− 0.62 (0.65) − 0.50 (− 3.00 to 0.00)	− 0.39 (0.47) − 0.25 (− 1.50 to 0.25)	− 0.10 (0.32) − 0.13 (− 0.96 to 0.77)	− 0.11 (0.28) 0.00 (− 1.15 to 0.64)	0.57 (0.50) 0.35 (0.00 to 2.12)	0.03 (0.07) 0.00 (0.00 to 0.30)
5 years	37	0.08 (0.11) 0.05 (0.00 to 0.40)	− 0.14 (0.41) 0.00 (− 1.00 to 1.00)	− 0.65 (0.64) − 0.50 (− 3.00 to 0.00)	− 0.46 (0.51) − 0.25 (− 1.50 to 0.25)	− 0.10 (0.33) 0.00 (− 0.96 to 0.70)	− 0.12 (0.28) 0.00 (− 1.15 to 0.48)	0.64 (0.51) 0.35 (0.00 to 2.12)	0.04 (0.08) 0.00 (0.00 to 0.30)
6 years	36	0.08 (0.12) 0.05 (0.00 to 0.40)	− 0.13 (0.42) 0.00 (− 1.00 to 1.00)	− 0.65 (0.62) − 0.50 (− 3.00 to 0.00)	− 0.46 (0.52) − 0.25 (− 1.50 to 0.50)	− 0.09 (0.33) − 0.09 (− 0.96 to 0.70)	− 0.12 (0.27) − 0.04 (− 1.15 to 0.48)	0.65 (0.50) 0.35 (0.00 to 2.12)	0.04 (0.08) 0.00 (0.00 to 0.30)
7 years	34	0.09 (0.12) 0.05 (0.00 to 0.40)	− 0.10 (0.39) 0.00 (− 1.25 to 1.00)	− 0.62 (0.54) − 0.50 (− 2.00 to 0.00)	− 0.46 (0.49) − 0.25 (− 1.50 to 0.50)	− 0.09 (0.30) − 0.18 (− 0.77 to 0.70)	− 0.13 (0.24) − 0.04 (− 0.87 to 0.48)	0.64 (0.45) 0.35 (0.00 to 1.58)	0.04 (0.08) 0.00 (0.00 to 0.30)
8 years	27	0.10 (0.12) 0.05 (0.00 to 0.40)	− 0.16 (0.42) 0.00 (− 1.25 to 1.00)	− 0.68 (0.54) − 0.50 (− 2.00 to 0.00)	− 0.56 (0.52) − 0.50 (− 1.50 to 0.50)	− 0.10 (0.31) − 0.19 (− 0.77 to 0.70)	0.25 (0.24) 0.25 (0.00 to 0.94)	0.77 (0.46) 0.61 (0.00 to 1.66)	0.05 (0.09) 0.00 (0.00 to 0.30)
9 years	27	0.11 (0.13) 0.05 (0.00 to 0.40)	− 0.18 (0.42) 0.00 (− 1.25 to 1.00)	− 0.68 (0.54) − 0.50 (− 2.00 to 0.00)	− 0.57 (0.51) − 0.50 (− 1.50 to 0.50)	− 0.10 (0.31) − 0.19 (− 0.77 to 0.70)	0.25 (0.24) 0.25 (0.00 to 0.94)	0.78 (0.44) 0.61 (0.25 to 1.66)	0.05 (0.09) 0.00 (0.00 to 0.30)
10 years	24	0.07 (0.13) 0.07 (0.00 to 0.40)	− 0.25 (0.44) − 0.25 (− 1.25 to 1.00)	− 0.72 (0.49) − 0.50 (− 2.00 to 0.00)	− 0.59 (0.52) − 0.50 (− 1.62 to 0.50)	− 0.13 (0.23) − 0.07 (− 0.74 to 0.47)	− 0.16 (0.25) − 0.09 (− 0.87 to 0.32)	0.75 (0.45) 0.56 (0.25 to 1.63)	0.05 (0.09) 0.00 (0.00 to 0.30)
11 years	17	0.12 (0.13) 0.10 (0.00 to 0.40)	− 0.26 (0.47) − 0.25 (− 1.25 to 1.00)	− 0.74 (0.36) − 0.50 (− 1.50 to − 0.50)	− 0.61 (0.51) − 0.50 (− 1.75 to 0.50)	− 0.08 (0.19) − 0.09 (− 0.54 to 0.24)	0.24 (0.19) 0.25 (0.00 to 0.70)	0.75 (0.44) 0.61 (0.13 to 1.89)	0.06 (0.09) 0.05 (0.00 to 0.30)
12 years	11	0.14 (0.14) 0.10 (0.00 to 0.40)	− 0.27 (0.58) − 0.25 (− 1.25 to 1.00)	− 0.89 (0.38) − 1.00 (− 1.50 to − 0.50)	− 0.72 (0.58) − 0.75 (− 1.75 to 0.50)	− 0.02 (0.39) − 0.09 (− 0.57 to 0.70)	− 0.23 (0.21) − 0.25 (− 0.49 to 0.19)	0.95 (0.40) 0.90 (0.39 to 1.82)	0.06 (0.10) 0.05 (0.00 to 0.30)
13 years	6	0.14 (0.15) 0.10 (0.00 to 0.40)	− 0.17 (0.58) − 0.25 (− 0.75 to 0.75)	− 0.79 (0.40) − 0.63 (− 1.50 to − 0.50)	− 0.56 (0.54) − 0.75 (− 1.00 to 0.25)	0.25 (0.31) 0.25 (− 0.22 to 0.74)	− 0.11 (0.19) − 0.13 (− 0.38 to 0.22)	0.80 (0.34) 0.79 (0.39 to 1.25)	0.07 (0.11) 0.05 (0.00 to 0.30)

SD: standard deviation; D: diopters; UDVA: uncorrected distance visual acuity; SE: spherical equivalent; J_0_ and J_45_: power vectors of refractive astigmatism; B: overall blur strength; CDVA: corrected distance visual acuity

**Fig. 2:**
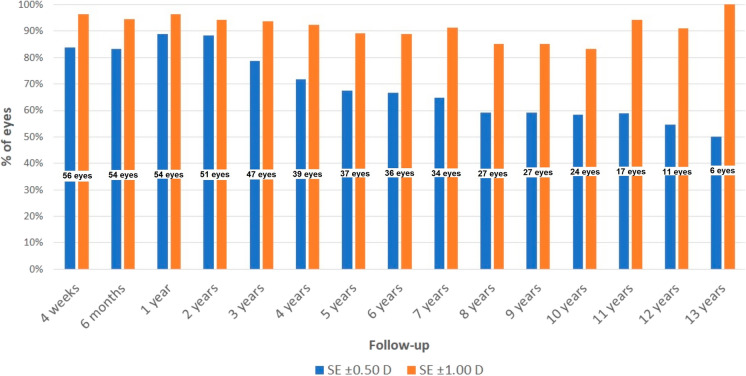
Percentage of eyes with a spherical equivalent (SE) within ± 0.50 and ± 1.00 D during the whole follow-up

Figure 2 shows the evolution during the whole follow-up of the percentage of eyes with SE within ± 0.50 D and ± 1.00 D. As shown, the percentage of eyes with SE within ± 1.00 D was maintained over a value of 83% during the whole follow-up. In contrast, a trend to a reduction in the percentage of eyes with SE within ± 0.50 D was observed in the long term.

### Intraocular pressure (IOP) changes

**Fig. 3:**
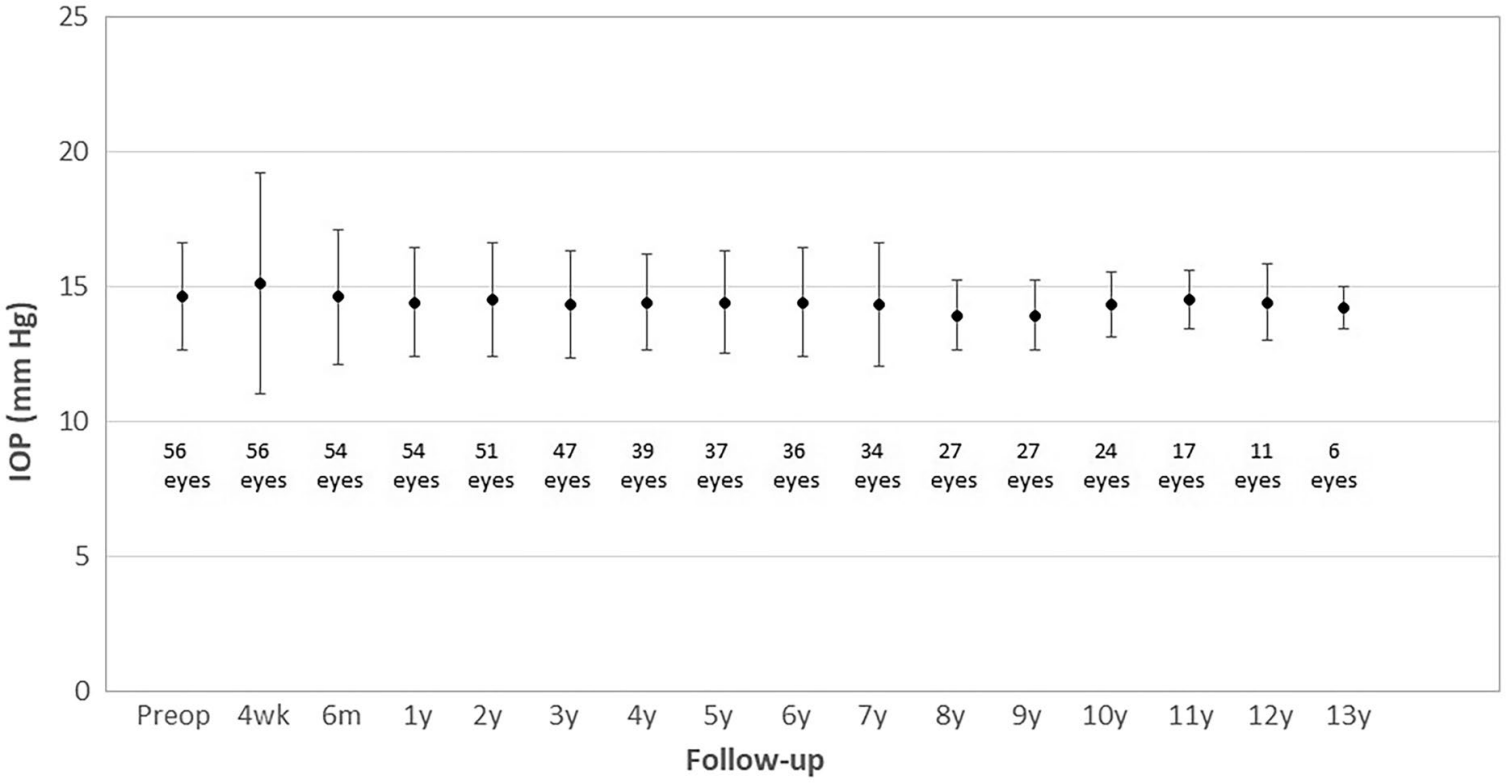
Changes in intraocular pressure (IOP) during the follow-up (preop, preoperative; wk, week; m, month; y, year)

Figure 3 shows changes in mean IOP during the whole follow-up. As shown, this parameter experienced small changes in the long term, although some of them did reach statistical significance (*p* < 0.001). There was a non-significant trend to an increase in IOP in the initial postoperative period (preop-4 weeks postop, *p* = 0.530), with a significant reduction afterwards (4 weeks–1 year postop, *p* = 0.039). No significant changes in IOP were detected during the rest of follow-up (1–13 years postop, *p* = 0.180).

### Anterior chamber depth (ACD) changes

**Fig. 4:**
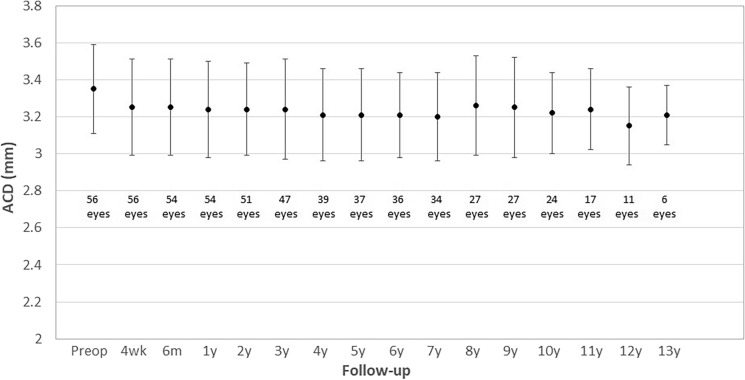
Changes in anterior chamber depth (ACD) during the follow-up (preop, preoperative; wk, week; m, month; y, year)

Figure 4 shows changes in mean ACD during the whole follow-up. As shown, this parameter also experienced changes of low magnitude in the long term, although some of them reached statistical significance (*p* < 0.001). There was an initial significant reduction of ACD at 4 weeks after surgery (*p* < 0.001), with no significant changes during the following 9 years of follow-up (*p* = 0.118). Finally, between 10 and 13 years postoperatively, an additional statistically significant reduction in ACD was observed (*p* = 0.027).

### Endothelial cell density (ECD) changes

**Fig. 5:**
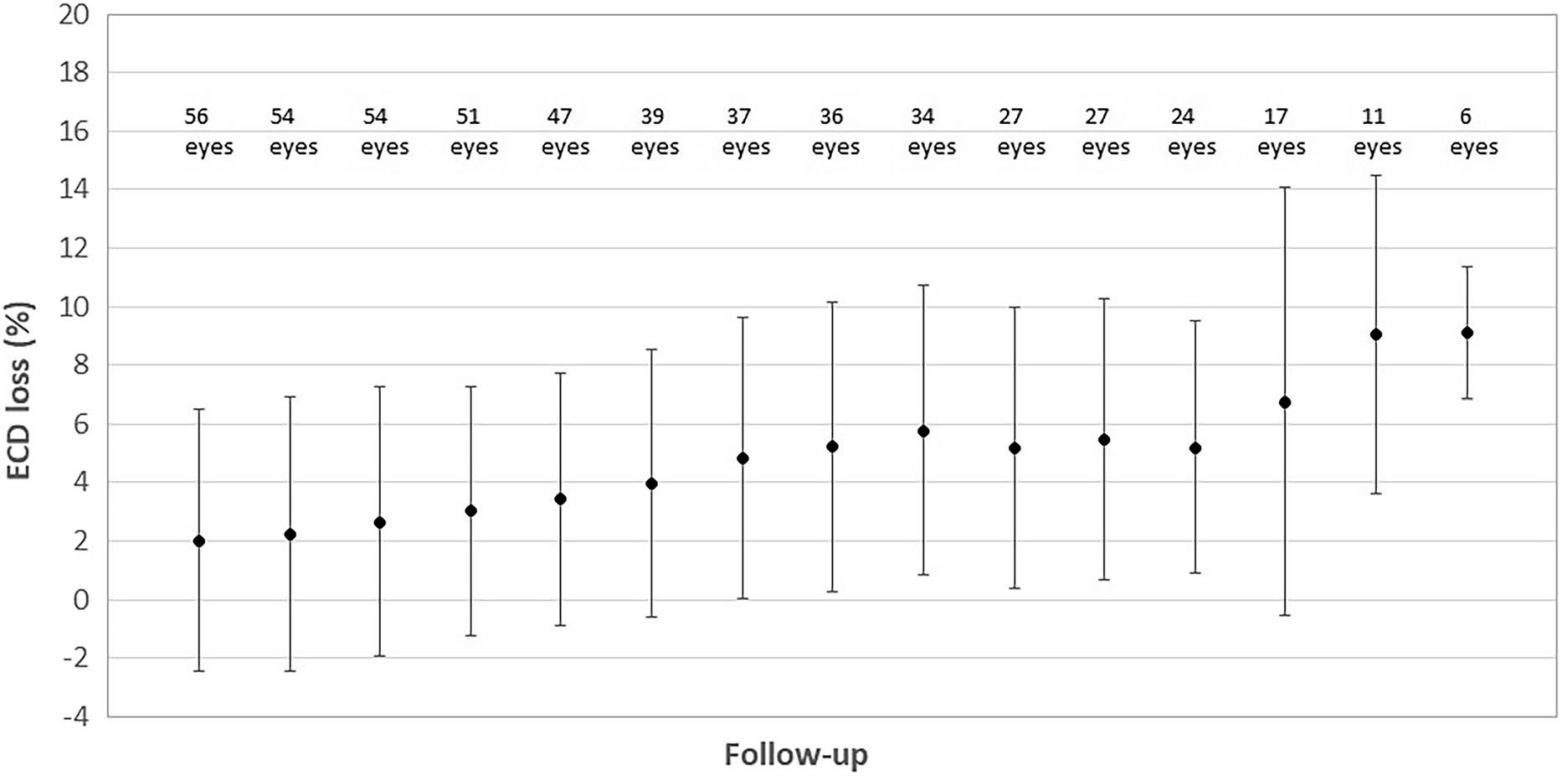
Percentage of loss of endothelial cell density (ECD) during the follow-up (preop, preoperative; wk, week; m, month; y, year)

Table 3 summarizes the endothelial cell data of the sample evaluated during the whole follow-up. Significant changes were found in ECD during the follow-up (*p* < 0.001). At 4 weeks after surgery, this parameter experienced a significant reduction (*p* < 0.001), with additional significant reductions during the rest of follow-up (*p* = 0.028). The mean endothelial cell loss changed from 2.01 ± 4.49% at 4 weeks after surgery to 9.11 ± 2.24% at the end of the follow-up (13 years) (Fig. 5). Changes in the standard deviation of the analysed endothelial cell dimensions (SD EC) during the whole follow-up did not reach statistical significance (*p* = 0.204). Regarding the coefficient of variation of the analysed endothelial cells (CV), it varied significantly during the follow-up (*p* = 0.002). Specifically, the change in CV in the early postoperative period was not significant (*p* = 0.081), but the reduction of this parameter in the long term did reach statistical significance (*p* = 0.044).

**Table 3:** Summary of endothelial cell data in the sample evaluated

Mean (SD) Median (Range)	N	Endothelial cell density (cell/mm^2^)	% of change of endothelial cell density	SD EC (mm^2^)	CV (%)
Preoperative	56	2829.46 (204.00) (2447 to 3409)	–	29.48 (5.92) (13 to 40)	34.34 (10.08) (9 to 55)
weeks	56	2771.36 (224.49) (2357 to 3345)	2.01 (4.49) 1.55 (− 9.13 to 17.38)	28.96 (5.31) (12 to 45)	32.46 (10.48) (9 to 54)
6 months	54	2776.15 (221.61) (2350 to 3341)	2.22 (4.69) 1.74 (− 8.89 to 17.51)	28.76 (5.20) (12 to 43)	32.19 (10.73) (9 to 54)
1 year	54	2763.89 (212.19) (2347 to 3218)	2.64 (4.60) 2.09 (− 8.74 to 17.61)	29.07 (5.38) (12 to 42)	32.48 (10.40) (9 to 54)
2 years	51	2758.49 (195.09) (2344 to 3149)	3.01 (4.23) 3.10 (− 6.09 to 17.65)	29.63 (5.01) (20 to 42)	32.57 (9.65) (13 to 53)
3 years	47	2743.72 (198.51) (2339 to 3141)	3.42 (4.29) 3.27 (− 4.89 to 17.78)	30.02 (4.76) (21 to 42)	32.23 (9.71) (13 to 53)
4 years	39	2745.13 (194.25) (2335 to 3061)	3.97 (4.58) 3.74 (− 4.75 to 18.43)	30.05 (4.93) (21 to 42)	31.87 (9.23) (13 to 53)
5 years	37	2711.59 (185.59) (2329 to 3058)	4.83 (4.78) 4.44 (− 4.65 to 19.08)	30.03 (4.80) (21 to 42)	32.41 (9.18) (13 to 53)
6 years	36	2694.67 (189.51) (2320 to 3046)	5.22 (4.94) 4.67 (− 4.26 to 19.18)	29.94 (4.58) (21 to 41)	32.33 (9.05) (13 to 53)
7 years	34	2664.91 (183.06) (2317 to 3043)	5.78 (4.94) 5.28 (− 4.14 to 19.41)	29.91 (4.49) (21 to 41)	32.00 (9.12) (13 to 53)
8 years	27	2649.74 (192.19) (2230 to 3039)	5.19 (4.81) 5.38 (− 4.82 to 19.41)	29.63 (4.96) (21 to 41)	30.48 (9.78) (13 to 51)
9 years	27	2642.37 (194.32) (2228 to 3036)	5.46 (4.78) 5.65 (− 4.75 to 19.41)	29.48 (4.96) (21 to 41)	30.15 (9.75) (13 to 51)
10 years	24	2636.04 (147.41) (2361 to 2888)	5.18 (4.31) 5.72 (− 14.06 to 8.88)	28.88 (5.07) (21 to 41)	28.50 (9.53) (12 to 50)
11 years	17	2597.35 (138.03) (2359 to 2830)	6.76 (7.31) 7.50 (− 13.34 to 23.06)	28.94 (5.39) (22 to 40)	27.94 (10.88) (12 to 49)
12 years	11	2579.18 (138.55) (2358 to 2825)	9.06 (5.44) 7.91 (1.35 to 23.09)	28.36 (5.41) (22 to 39)	27.36 (7.23) (18 to 42)
13 years	6	2602.17 (132.59) (2447 to 2823)	9.11 (2.24) 8.21 (7.21 to 13.12)	29.33 (5.96) (22 to 39)	29.33 (8.87) (18 to 42)

SD: standard deviation; SD EC: standard deviation of the analyzed endothelial cell dimensions; CV: coefficient of variation of the analyzed endothelial cells, derived by dividing the average dimension by the standard deviation

### Complications

No complications were reported during the follow-up and the explantation of the phakic IOL was not required in any case.

## Discussion

In the current study, a comprehensive analysis of the ocular and visual changes occurring in a long-term follow-up in patients implanted with the pIOL Artiflex has been performed. These studies are critical to define the safety of these intraocular implants as some pIOL models have been associated to some complications in the long term that should have been considered prior to their recommendation [19]. Specifically, concerning the foldable iris-fixated pIOL evaluated in the current study, some previous studies have been conducted confirming that the integrity of corneal endothelium is preserved and that complications are minimal [1–3, 8]. The aim of the current study was to analyse retrospectively the clinical outcomes obtained during a long-term follow-up period with the Artiflex pIOL, evaluating the efficacy and stability of the refractive correction achieved as well as the safety profile of the pIOL (endothelial cell changes, potential shortening of the anterior chamber, IOP changes and complications).

As demonstrated in many previous studies, this pIOL allows an effective refractive correction and the corresponding visual rehabilitation, leading to complete spectacle independence [1–15]. Specifically, in our long-term series, a significant reduction of refractive error with a significant improvement of UDVA associated was observed at 4 weeks after surgery, with 83% of patients or more maintaining during the 13-year follow-up a SE within ± 1.00 D. Similarly, Papa-Vetorazzi et al. [2] found in a sample of 70 eyes implanted with the Artiflex pIOL and followed for more than 10 years that 61% and 76% of eyes were within ± 0.50 D and ± 1.00 D of attempted SE correction at the end of the follow-up, respectively. Despite the non-significant changes in sphere, SE and UDVA during the follow-up in our series, there was a slight trend to myopization over time. Likewise, there was a significant change in the long-term in the J_0_ component of refractive astigmatism. This trend does not seem to be related to the implant behaviour as its stability and biocompatibility was confirmed at all yearly visits of the follow-up. This may be related to progressive changes in axial length and corneal curvature associated to increasing age. It has been shown that myopia progression continues for more than one-third of adults during the third decade of life, albeit at lower rates than during childhood [20]. Likewise, some age-related changes in ocular astigmatism have been described due to alterations in the position and tension of the eyelids, corneal stromal collagen fibrils, Descemet membrane, and extraocular muscles [21].

In our sample, there were no losses of CDVA during the follow-up. This could be explained by the good optical performance of the implant as well as the lack of any complication affecting the retina or intraocular media. Papa-Vetorazzi et al. [2] only found that 4% of eyes lost 1 line of CDVA in their long-term series evaluating the same pIOL, with any patient losing two lines. They only reported during the follow-up 1 traumatic dislocation requiring re-enclavation at 8.7 years postoperatively. In another study conducted by Monteiro et al. [4] with a 6-year follow-up after implantation of the Artiflex pIOL, a total of 5.7% of eyes lost 1 or more lines of CDVA. Likewise, Marta et al. [1] in another series evaluating the clinical outcomes of the same pIOL did not find significant changes in CDVA during a follow-up of 10 years or more. Therefore, the pIOL evaluated is safe in terms of preserving the level of vision of the patient, with minimal incidence of complications associated.

Concerning IOP, there was a non-significant trend to its increase in the initial postoperative period (4 weeks postoperatively), and a significant reduction afterwards from the 4-week to the 1-year postoperative visit. Similarly, no significant increases in IOP have been also reported in other long-term series evaluating the outcomes of the Artiflex pIOL [1, 2]. This is consistent with the maintenance of a wide anterior chamber after surgery and a minimal reduction of ACD. Despite the expected initial reduction of the ACD with the implantation of the pIOL (mean change at 4 weeks = −0.10 ± 0.09 mm), no significant changes were observed during the following nine years of follow-up. However, an additional decrease of ACD was observed during the last 3 years of the follow-up (mean change = −0.04 ± 0.04 mm). This variation is consistent with age-related physiological ACD changes due to the thickening of the crystalline lens.

Finally, corneal endothelial cell changes were also evaluated, with an ECD loss of 2.01 ± 4.49% at 4 weeks after surgery that increased to 9.11 ± 2.24% at the end of the follow-up (13 years). This ECD loss is consistent with those reported in other long-term series evaluating the performance of the same pIOL [1, 2]. Papa-Vetorazzi et al. [2] found in a sample of eyes implanted with the Artiflex pIOL and followed for more than 10 years a mean ECD loss of 12.2 ± 12.5%. Considering that in the healthy eye the physiological cell loss rate over a 10-year period is around 0.6% per year [22], an ECD loss of around 8% could be expected during a period of 13 years only due to age-related changes. According to this estimation, the ECD loss rate found with the pIOL evaluated was then on average 1.11% higher than the physiological rate. Considering that ACD was maintained over a considerable amount of years and the absence of complications, this slightly increased ECD loss rate could be mainly attributed to the surgical trauma as a mean loss of 2.01% was found after the first 4 weeks of the surgical procedure. Besides ECD, other aspects of corneal endothelium were evaluated, including the standard deviation (SD EC) and coefficient of variation (CV) of the analysed endothelial cell dimensions. No significant changes were found during the follow-up in SD EC, and changes in CV were of low magnitude although statistically significant. It should be considered that the age-related ECD loss is also associated with an increase in the CV and consequently in the level of polymegathism [22]. In summary, corneal endothelial changes found in the current long-term series confirm the safety of the implant in this context, as in previous studies [1, 2, 8].

This retrospective series has some weaknesses and several limitations that must be mentioned. It is a long-term study but fortunately the same devices and the same clinicians were involved in the study during the whole follow-up and consequently inter-device and inter-observer biases are absent. However, not all patients reached the end period of the follow-up as they were operated on afterwards and the follow-up was still ongoing. This led to the limitation of the sample size at the last visits of the follow-up. Furthermore, both eyes of some patients were included and this may have introduced some level of bias. However, despite this, significant changes were detected and some trends were identified that should be confirmed in future studies with larger samples and even longer follow-ups. One strength of this study that must be mentioned is that the parameters at more time points are reported (data was collected and reported annually after the first year) when compared to other long-term studies, such as those from Marta et al. [1] and Papa-Vettorazzi et al. [2].

In conclusion, the myopia correction with the spherical model of the Artiflex phakic intraocular lens is an effective and safe procedure in the long term, with minimal anatomical changes that could be mainly attributed to age-related processes and a stable position within the anterior chamber not leading to complications.
